# Cyclooxygenase-2 Inhibition Attenuates Abdominal Aortic Aneurysm Progression in Hyperlipidemic Mice

**DOI:** 10.1371/journal.pone.0044369

**Published:** 2012-11-27

**Authors:** Sarbani Ghoshal, Charles D. Loftin

**Affiliations:** Department of Pharmaceutical Sciences, College of Pharmacy, University of Kentucky, Lexington, Kentucky, United States of America; Maastricht University, The Netherlands

## Abstract

Abdominal aortic aneurysms (AAAs) are a chronic inflammatory disease that increase the risk of life-threatening aortic rupture. In humans, AAAs have been characterized by increased expression of cyclooxygenase-2 and the inactivation of COX-2 prior to disease initiation reduces AAA incidence in a mouse model of the disease. The current study examined the effectiveness of selective cyclooxygenase-2 (COX-2) inhibition on reducing AAA progression when administered after the initiation of AAA formation. AAAs were induced in hyperlipidemic apolipoprotein E-deficient mice by chronic angiotensin II (AngII) infusion and the effect of treatment with the COX-2 inhibitor celecoxib was examined when initiated at different stages of the disease. Celecoxib treatment that was started 1 week after initiating AngII infusion reduced AAA incidence by 61% and significantly decreased AAA severity. Mice treated with celecoxib also showed significantly reduced aortic rupture and mortality. Treatment with celecoxib that was started at a late stage of AAA development also significantly reduced AAA incidence and severity. Celecoxib treatment significantly increased smooth muscle alpha-actin expression in the abdominal aorta and did not reduce expression of markers of macrophage-dependent inflammation. These findings indicate that COX-2 inhibitor treatment initiated after formation of AngII-induced AAAs effectively reduces progression of the disease in hyperlipidemic mice.

## Introduction

Abdominal aortic aneurysms (AAAs) are a chronic condition that often begin as minor vessel dilation that progresses over years to produce a weakened aorta with increased susceptibility for rupture. Although AAAs at all stages of progression are readily detectable in humans by noninvasive imaging techniques, there are no pharmacological treatments currently available to slow progression at an early stage of the disease or cause regression of fully-formed aneurysms. Approximately 80% of the small aneurysms that are detected eventually require surgical repair, which is currently the only known successful form of treatment [1], [2]. However, because of significant risk associated with surgical repair, this treatment is considered acceptable only for those patients diagnosed with the most severe form of the disease. The characterization of mechanisms contributing to aortic aneurysmal remodeling will facilitate identification of medications that are effective for reducing progression when treatment is initiated after diagnosis of the disease.

We previously identified cyclooxygenase-2 (COX-2) that is expressed in smooth muscle cells (SMCs) of the abdominal aorta as an important contributor to AAA development in a mouse model of the disease. We showed that AAA incidence and severity that was induced in mice by chronic infusion of angiotensin II (AngII) were significantly reduced by pre-treatment with the COX-2 inhibitor celecoxib or by targeted genetic inactivation of COX-2 prior to initiating AngII infusion [3], [4]. The effectiveness of COX-2 inactivation prior to disease initiation was associated with a reduction in macrophage-dependent inflammation in the abdominal aorta at an early stage of the disease [4]. The inactivation of microsomal prostaglandin E synthase-1 (mPGES-1) in mice prior to initiation of the disease also results in reduced AAA incidence and severity [5]. However, the mechanism for the effectiveness of mPGES-1 deficiency does not result from attenuated macrophage-dependent inflammation. Because mPGES-1 is primarily thought to function down-stream of COX-2, the proinflammatory effects of COX-2-derived prostanoids that function at an early stage of AAA formation may be distinct from COX-2-dependent mechanisms contributing to later stage AAA progression. In the current study, we report that inhibition of COX-2 with celecoxib shows dramatic effectiveness for reducing AAA progression and aortic rupture when treatment is first started well after initiation of the disease. The effectiveness of COX-2 inhibition is not associated with attenuated macrophage-dependent inflammation, but does correlate with characteristics of increased SMC differentiation.

## Experimental Procedures

### Animals

ApoE-deficient male mice on a C57BL/6J background strain purchased from the Jackson Laboratories (Bar Harbor, Maine) were allowed to age from 14–16 weeks before beginning each study. For the studies that utilized an AngII infusion of 6 weeks, osmotic pumps were implanted in mice at 4 months of age and studies with an 8-week AngII infusion utilized 3.5 month-old mice. Mice were housed under barrier conditions with food and water *ad libitum*. For subcutaneous osmotic pump implantation, mice were anesthetized with isoflurane at a concentration of 2% in oxygen that was delivered using a precision vaporizer and an induction box. For performing euthanasia, mice were first anesthetized by intraperitoneal injection of ketamine (100 mg/kg) and xylazine (10 mg/kg) until unresponsive to pain, followed by thoracotomy and exsanguination under anesthesia. All studies were conducted under the approval of the University of Kentucky Institutional Animal Care and Use Committee (approval No. 2008-0262) and conform to the Guide for the Care and Use of Laboratory Animals published by the National Institutes of Health (NIH Publication No. 85–23, revised 1996).

### AngII Infusion

Mice were implanted with subcutaneous osmotic pumps (Alzet model 2004 28-day delivery or model 2006 42-day delivery) containing AngII (Sigma). AngII was infused at a rate of 1 µg/kg/min for the 6-week infusion studies and 0.75 µg/kg/min for the 8-week AngII infusions. For the 8-week AngII infusions, 28-day delivery pumps (model 2004) were replaced with new pumps after the first 4 weeks of infusion. The 0.75 µg/kg/min AngII infusion rate for the 8-week study was used to allow for an acceptable level of mortality during this prolonged continuous exposure to AngII.

### Celecoxib Administration

Mice were provided normal laboratory diet (Harlan Teklad) prior to replacing with a pelleted diet containing 1000 ppm celecoxib (LKT Laboratories Inc.) or a matched control diet (Purina 5002) that was pelleted without celecoxib (Research Diets, Inc.). The 1000 ppm dose of celecoxib added to the diet has been shown to not alter the weight of the mice or affect blood pressure following AngII infusion [3]. We have reported previously, that the 1000 ppm celecoxib concentration in the diet results in a plasma concentration of approximately 1.6 µg/ml, which approximates the therapeutic plasma concentration of celecoxib in humans [3], [6]. For the study analyzing the effect of celecoxib treatment beginning at early-stage of AAA progression, mice were infused with 1 µg/kg/min of AngII, as described above, and after 1 week were either provided control diet or diet containing celecoxib. The mice were then sacrificed for analysis after 6 weeks of AngII infusion. For the study determining the effect of celecoxib treatment beginning at late-stage AAA progression, mice were infused with 0.75 µg/kg/min of AngII, as described above, and after 3 weeks were either provided control diet or diet containing celecoxib, followed by analysis after 8 weeks of AngII infusion.

### Vascular Pathology

Following euthanasia, abdominal aortas of the mice were surgically exposed for analysis. Aneurysms in the supra-renal abdominal aorta were quantified by measuring the external diameter using a dissecting microscope and a digital caliper micrometer. Using this method of analysis under magnification, aneurysmal pathology was readily detectable with an abdominal aorta external diameter of 1.2 mm. Thus, the minimal diameter of 1.2 mm was used to designate the presence of an AAA. AAA severity was determined as a measurement of the external diameter and classified as Type 1: 1.2–2 mm or Type 2: 2–3 mm. Rupture of the aorta was defined by the identification of hematomas outside of the aorta in euthanized moribund mice or following necropsy of dead mice. Similar to our previous studies, mice that died prior to completion of the study as a result of aortic rupture were not included in the AAA incidence and severity calculations [4].

### Quantitation of mRNA Expression

Following euthanasia of the mice, the abdominal segment of the aorta containing the region distal of the intercostal arteries to below the renal arteries was excised and stored in RNALater (Sigma). Total RNA was extracted from the abdominal segments using RNeasy Mini kits (Qiagen). Quantitative gene expression was performed in a two-step RT-PCR using Superscript II (Invitrogen) and fluorogenic 5′ nuclease chemistry (Taqman Assays, Applied Biosystems). A ratio of expression level between the mRNA of interest and the constitutively expressed housekeeping gene hypoxanthine phospho-ribosyl transferase (HPRT) was quantified using manufacturers recommended PCR conditions (Applied Biosystems) [4], [7], [8]. A standard curve from within each PCR run was used for the relative standard curve method of quantitating mRNA expression.

### Histological Analysis

Following euthanasia, cardiac puncture was performed to perfuse the aorta with PBS and then 10% neutral buffered formalin solution prior to collection for histological analysis. Suprarenal abdominal aortas were processed in a dehydrating ethanol gradient, followed by xylene incubation and paraffin embedding. Eight micron sections were used for hematoxylin and eosin (H&E) staining and immunohistochemical analysis of COX-2 (primary antibody from Cayman Chemical) or smooth muscle α-actin (Dako Cytomation). Hyaluronic acid was detected using biotinylated hyaluronic acid binding protein (Calbiochem). Antibodies or hyaluronic acid binding protein was detected using Vectastain Elite ABC kit (Vector Laboratories) and di-amino benzidine staining using manufacturer's instructions. Immunohistochemical sections were counterstained with hematoxylin.

### Statistical Analyses

For analysis of AAAs and mRNA expression in the abdominal aorta, tissue isolated from one individual mouse was considered as an *n* of 1. Significant differences in incidence among different groups was determined using Fisher's exact test (Prism, GraphPad Software Inc.). The mean and SEM were determined for aortic diameter and mRNA expression and significant differences among groups were determined using unpaired two-tailed *t*-test, with differences being considered statistically significant at *P*<0.05.

## Results

### COX-2 inhibitor treatment initiated during early-stage AAA progression reduces AAA incidence

**Figure 1:**
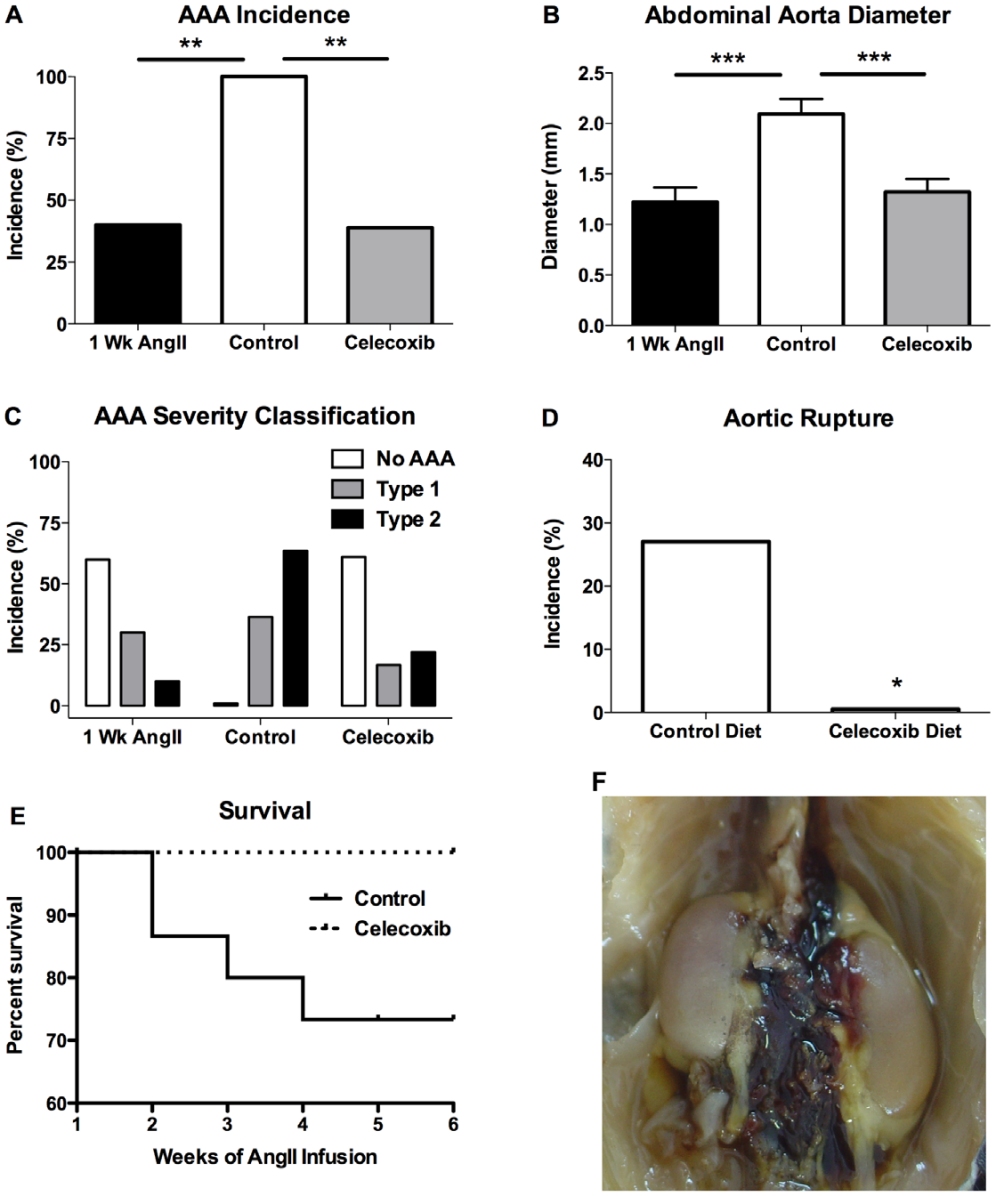
COX-2 inhibitor treatment initiated 1 week after beginning AngII infusion effectively reduces AAA progression in ApoE-deficient mice. (A) AAA incidence was determined at necropsy following 1 (1 Wk AngII) or 6 weeks (Control and Celecoxib) of AngII infusion. (B) The maximal external diameter or (C) AAA classification of the suprarenal aorta following 1 (1 Wk AngII) or 6 weeks (Control and Celecoxib) of AngII infusion. (D) Incidence of aortic rupture in mice on control or celecoxib containing diet following 6 weeks of AngII infusion. (E) Survival of Control and Celecoxib-treaded mice during the AngII infusion. (F) Representative image of postmortem detection of hemorrhage in the abdominal cavity indicating aortic rupture. For aortic diameter, results depict mean ± SEM. AAA incidence and diameter: 1 Wk AngII *n* = 10, Control *n* = 11, Celecoxib *n* = 18; aortic rupture: Control *n* = 15, Celecoxib *n* = 18. *P* values were determined for incidence data using Fisher's exact test and an unpaired two-tailed *t*-test for aortic diameter. * indicates *P*<0.05, ** indicates *P*<0.01, *** indicates *P*<0.001.

The infusion of AngII for 1 week in mice not treated with celecoxib resulted in a 40% incidence of AAAs (Figure 1A). As compared to the 1-week infusion, the AAA incidence was significantly increased in control mice receiving AngII infusion for 6 weeks (Figure 1A). For the celecoxib-treated mice, the COX-2 inhibitor was started 7 days following the initiation of AngII infusion and was continued throughout the remaining 5 weeks of the infusion. As shown in Figure 1A, the 5 weeks of celecoxib treatment significantly reduced AAA incidence when analyzed after completion of the 6-week AngII infusion, as compared to control mice not receiving celecoxib. These findings suggest that beginning celecoxib treatment at an early-stage of the disease reduces the increase in AAA incidence that occurs with continued AngII infusion.

### COX-2 inhibitor treatment initiated during early-stage AAA progression reduces AAA severity

AngII-induced AAAs are characterized by expansion of the external diameter of the abdominal aorta [9]. The external diameter of the abdominal aorta was compared between mice infused with AngII for 1 week or 6 weeks. As shown in Figure 1B, the infusion of AngII for 6 weeks (Control) resulted in a significant increase in AAA size, as compared to mice infused with AngII for 1 week, suggesting significant expansion of AAA severity during the additional 5 weeks of the infusion. The administration of celecoxib during the final 5 weeks of AngII infusion significantly reduced the mean external diameter of the abdominal aorta, as compared to mice on control diet infused with AngII for 6 weeks (Figure 1B). Classification of the external diameter of the abdominal aorta showed reduced severity of the vascular pathology in mice receiving celecoxib treatment (Figure 1C). These findings indicate that celecoxib treatment is effective for reducing AAA severity when initiated 1 week after beginning the AngII infusion and suggests that the inhibition of COX-2 limits the progression of early AAAs once they have formed.

### COX-2 inhibitor treatment initiated during early-stage AAA progression reduces aortic ruptur

The AngII infusion model of AAA development is associated with a significant increase in mortality resulting from aortic rupture [10]. In the current study, the effect on aortic rupture and death was examined following celecoxib treatment that was initiated 1 week after beginning a 6-week AngII infusion. The incidence of aortic rupture and death that occurred during the final 5 weeks of the AngII infusion was significantly lower in the celecoxib treatment group, as compared to mice on control diet (Figure 1D). The mortality in the control group occurred from week 2 to week 4 of the AngII infusion (Figure 1E). Aortic rupture was determined by the detection of blood adjacent to the aorta following postmortem analysis of the thoracic and abdominal cavity (Figure 1F). These findings indicate that celecoxib treatment initiated 1 week after beginning the AngII infusion effectively reduced aortic rupture and mortality.

### COX-2 inhibitor treatment initiated during late-stage AAA progression reduces AAA incidence

**Figure 2:**
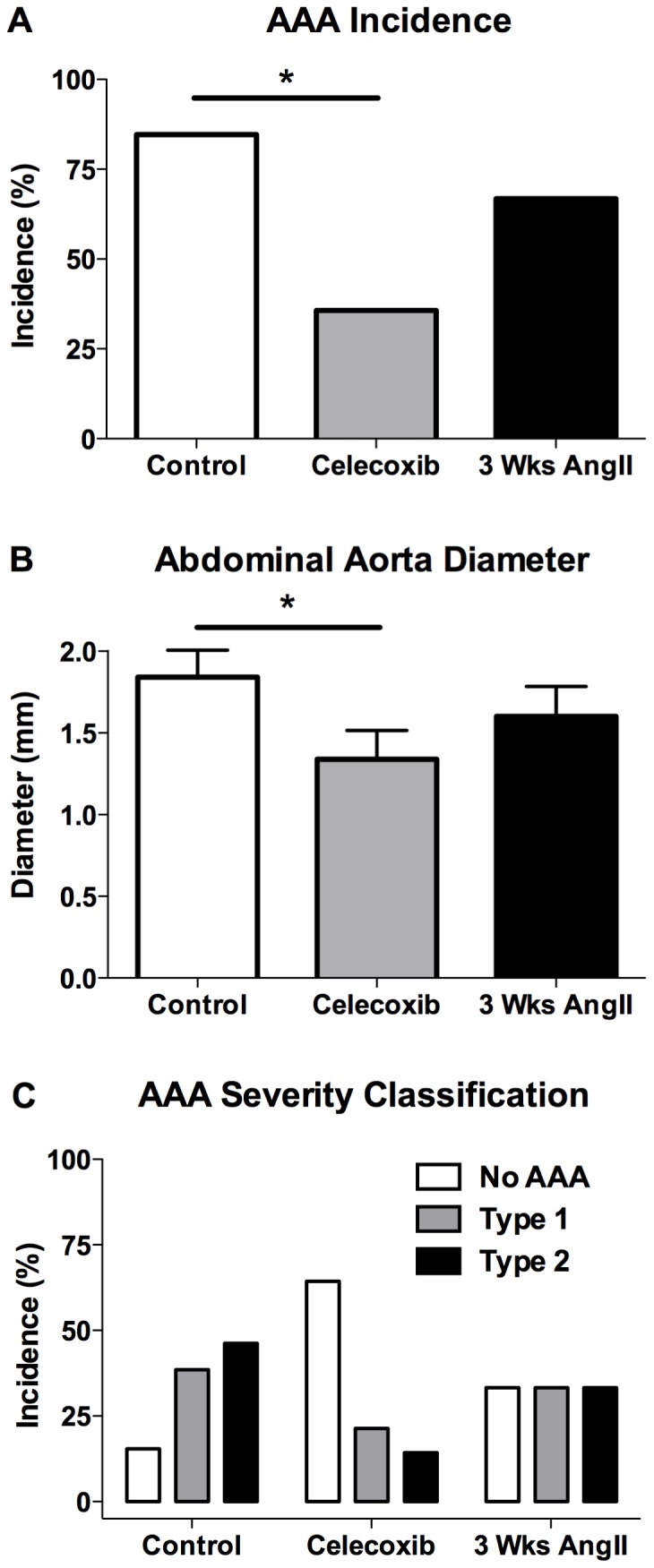
COX-2 inhibitor treatment initiated 3 weeks after beginning AngII infusion effectively reduces AAA progression in ApoE-deficient mice. (A) AAA incidence was determined at necropsy following 3 (3 Wks AngII) or 8 weeks (Control and Celecoxib) of AngII infusion. (B) The maximal external diameter or (C) AAA classification of the suprarenal aorta following 3 (3 Wks AngII) or 8 weeks (Control and Celecoxib) of AngII infusion. For aortic diameter, results depict mean ± SEM. AAA incidence and diameter: 3 Wks AngII *n* = 12, Control *n* = 13, Celecoxib *n* = 14. *P* values were determined for incidence data using Fisher's exact test and an unpaired two-tailed *t*-test for aortic diameter. * indicates *P*<0.05.

With our finding that celecoxib administration was effective when beginning treatment early after AAA initiation, we also examined celecoxib's effect on fully-formed AAAs. For celecoxib-treated mice, drug administration was begun 3 weeks after initiating the AngII infusion, and the AngII infusion for both control diet and celecoxib diet groups was continued for a total of 8 weeks, at which time AAA development was examined. As shown in Figure 2A, the celecoxib-treated mice showed a significant reduction in AAA incidence, as compared to mice on control diet (36% vs. 85% in the control mice). In order to estimate the AAA incidence in mice at the time of beginning the celecoxib treatment, the abdominal aortas of a separate group of mice not treated with celecoxib were analyzed after 3 weeks of AngII infusion (Figure 2A). Although the difference was not statistically significant, the incidence of AAAs following 3 weeks of AngII infusion was 67% (3 Wks AngII), as compared to a 36% AAA incidence in mice that received an additional 5 weeks of celecoxib treatment (Figure 2A). These findings suggest that beginning celecoxib treatment after significant AAA formation has occurred is effective in reducing the incidence of disease.

### COX-2 inhibitor treatment initiated during late-stage AAA progression reduces AAA severity

We also examined the effect on abdominal aorta diameter that resulted from initiating celecoxib treatment after advanced AAA formation. Celecoxib treatment was initiated 3 weeks after beginning the AngII infusion and was continued for the remaining 5 weeks of the infusion. As shown in Figure 2B, celecoxib treatment significantly reduced the external diameter of the abdominal aorta and decreased the severity of the vascular pathology (Figure 2C), as compared to 8-week AngII-infused mice on control diet. The severity of aneurysmal pathology at the time of beginning celecoxib was estimated by analyzing aortic diameter in a separate group of untreated mice after a 3 week AngII infusion. The aortic diameter after 3 weeks of AngII infusion showed an intermediate level of severity between the control and celecoxib treatment groups (Figure 2 B–C). These findings indicate that celecoxib treatment initiated after the development of significant aneurysmal pathology effectively reduced or reversed the progression of disease severity.

### Effect of COX-2 inhibitor treatment on expression of markers of inflammation

**Figure 3:**
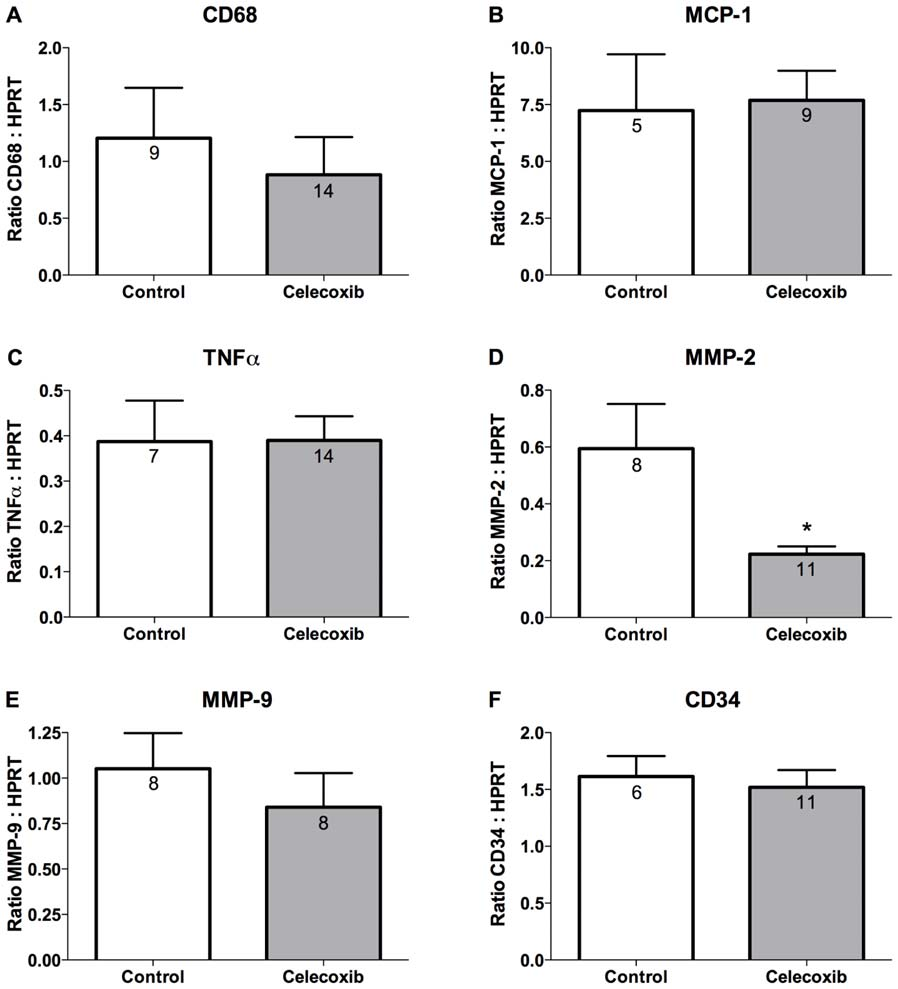
COX-2 inhibitor treatment does not alter macrophage-dependent inflammation. Abdominal aorta was collected at necropsy for control or celecoxib-treated mice beginning 1 week after initiating a 6-week AngII infusion. Real-time PCR quantitation of mRNA expression of (A) the macrophage marker CD68, (B) the monocyte recruitment chemokine MCP-1, (C) the proinflammatory cytokine TNFα, (D–E) metalloproteinases (MMPs) −2 and −9, and (F) the mast cell marker CD34. Expression levels of the gene of interest were normalized to HPRT mRNA levels. Results indicate mean ± SEM and the number of mice in each group is provided within the graphs. *P* values were determined using an unpaired two-tailed *t*-test. * indicates *P*<0.05.

We have previously shown that the initial stage of AAA development during the first week of AngII infusion is characterized by increased macrophage infiltration and increased expression of inflammatory cell recruitment chemokines [4]. In the current study, we examined the effect of celecoxib treatment initiated during early-stage AAA progression on macrophage-dependent inflammation in the abdominal aorta. Mice that were infused with AngII for 6 weeks and provided a diet without celecoxib were compared to mice that received celecoxib containing diet beginning 1 week after the initiation of a 6-week AngII infusion. The mRNA expression of neither the macrophage marker CD68, the monocyte recruitment chemokine MCP-1, nor the proinflammatory cytokine TNFα was significantly different in the abdominal aortas between control and celecoxib-treated mice (Figure 3 A–C). The macrophage-dependent inflammation that occurs following AAA formation has been shown to result in the increased expression of MMPs [1], [11], [12]. We examined the effect of celecoxib treatment on the mRNA expression of MMP-2 and MMP-9 in the abdominal aorta. Although the mRNA expression of MMP-2 was significantly reduced in the abdominal aorta of mice that received celecoxib (Figure 3D), there was no effect on the expression of MMP-9 (Figure 3E). In addition, there was no effect of celecoxib treatment on mRNA expression of CD34 (Figure 3F), which has been shown to be a marker of mast cells in mice [13]. These findings indicate that the effectiveness of COX-2 inhibition in reducing the progression of AAAs was not associated with reduced characteristics of inflammation.

### Increased expression of COX-2 is localized to SMCs in areas of aneurysmal remodeling

**Figure 4:**
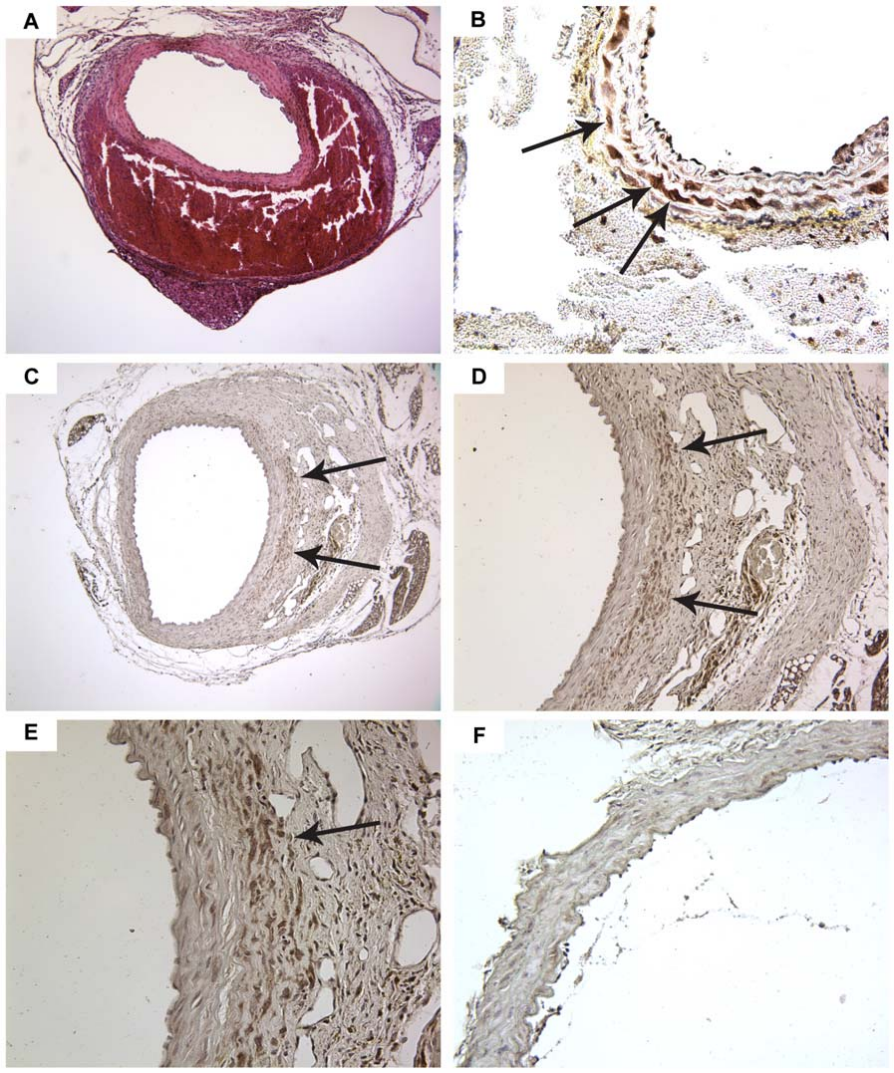
Histological analysis of the abdominal aorta during aneurysm progression. (A) Analysis of abdominal aorta containing a hematoma following histochemical staining by H&E. (B) Immunohistochemical analysis of COX-2 expression in abdominal aorta adjacent to hematoma. COX-2 expression in cells of the outer layers of a well-defined tunica media. Arrows indicate medial cells with concentrated expression of COX-2 surrounding individual nuclei. (C) COX-2 expression in abdominal aorta with adventitial aneurysmal remodeling. COX-2 expression is concentrated on the side of the aorta with aneurysmal remodeling without detectable expression on the non-involved side of the aorta (100× magnification). (D) COX-2 expression in cells just outside of the medial layer into a region between the media and the remodeled adventitia (200× magnification). (E) Cells with the greatest COX-2 expression are localized on the outer side of the external elastic lamina of the tunica media (400× magnification). (F) Immunohistochemical analysis of COX-2 expression in abdominal aorta without aneurysmal pathology (400× magnification). Arrows indicate regions with concentrated COX-2 expression. Brown staining from DAB indicates COX-2 and sections are counterstained with hematoxylin (blue).

With the lack of a role for COX-2-dependent inflammation, we examined the abdominal aorta microscopically to identify pathological changes in resident cells of the vascular wall during early aneurysm progression. The development of hematomas in the wall of the abdominal aorta occurs early in the course of AngII infusion [10]. In our current studies, we observed the occurrence of hematomas in the abdominal aorta prior to evidence of significant adventitial remodeling (Figure 4A). The regions of the abdominal aorta with evidence of a hematoma consistently showed the highest level of COX-2 expression, as determined by immunohistochemistry (Figure 4B). The cells with the greatest level of COX-2 expression were localized to the outer layer of smooth muscle adjacent to the hematoma (Figure 4B). In a subset of COX-2 expressing SMCs, a ring of concentrated expression around individual nuclei was evident (Figure 4B), indicating perinuclear expression, a known feature of COX-2 expression that is observed after exposure to potent inducers of COX-2 expression [14]. Therefore, at an early-stage of AAA development, COX-2 was highly expressed in SMCs of the outer medial layer prior to detection of aneurysmal remodeling.

The progression of AngII-induced AAAs results in the asymmetrical expansion of vascular wall remodeling that involves preferentially one side of the aorta [15]. Using immunohistochemistry, we examined COX-2 expression in the abdominal aorta of mice with early-stage aneurysmal remodeling. As shown in Figure 4C, COX-2 expression was primarily detected in cells adjacent to the aneurysmal pathology, rather than smooth muscle cells of the uninvolved aorta on the side of the vessel away from the AAA. The COX-2 expression was concentrated in multiple layers of disorganized cells outside of the media and in the remodeled adventitia (Figure 4D). The highest magnification shows elastic lamina between the medial layers with the greatest COX-2 expression being in cells outside of the external elastic lamina (Figure 4E). In contrast to the sections containing an AAA, COX-2 expression was not observed in the abdominal aorta of mice without evidence of aneurysmal remodeling (Figure 4F). These findings indicate that during aneurysmal progression COX-2 was preferentially expressed in cells involved in remodeling of the outer smooth muscle layer.

### SMCs involved in aneurysmal remodeling express characteristics of altered differentiation

**Figure 5:**
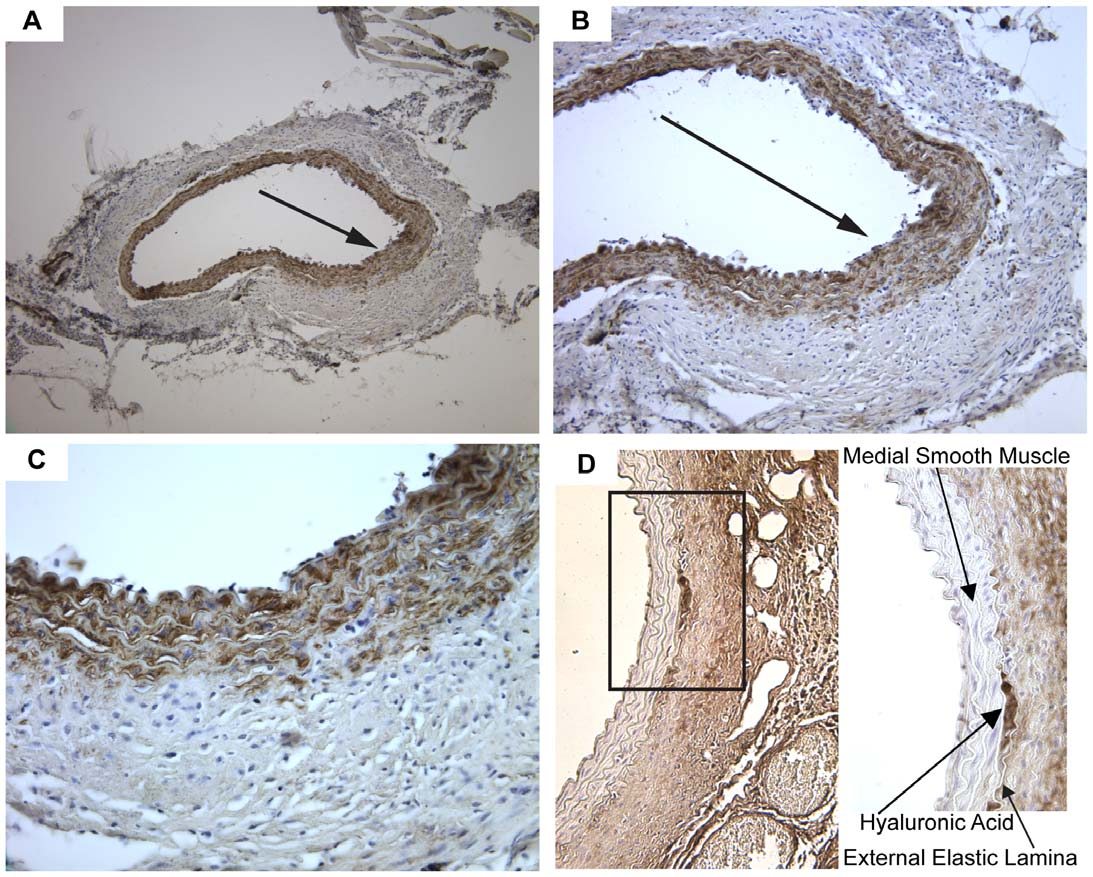
Immunohistochemical analysis of markers of smooth muscle cell differentiation and de-differentiation. (A) α-actin expressing smooth muscle cells localize to the side of the abdominal aorta involved in aneurysmal remodeling (100× magnification). (B) As compared to the non-involved aorta, α-actin expression is more diffuse (arrow) in the SMCs involved in aneurysmal remodeling (200× magnification). (C) α-actin expression is localized to SMCs within the medial layer and to SMCs outside of the external elastic lamina (400× magnification). (D) Hyaluronic acid expression is localized throughout the AAA and in cells within the medial side of the external elastic lamina. The boxed area is shown within the insert at 400× magnification. Brown staining from DAB indicates α-actin or hyaluronic acid detection and sections are counterstained with hematoxylin (blue).

The differentiated phenotype of smooth muscle cells of the medial layer has been defined by expression of contractile proteins. The contractile protein most often used to identify differentiated smooth muscle cells is smooth muscle α-actin, and is the most highly expressed actin in aortic smooth muscle [16]. We examined α-actin expression by immunohistochemistry in the abdominal aorta during AAA progression. In the abdominal aorta of mice with early-stage disease, aneurysmal pathology is primarily localized to one side of the aorta (Figure 5A). Although α-actin expression is observed throughout the medial layer of the aorta, the expression in cells involved in the aneurysmal remodeling is more diffuse than the concentrated expression observed on the uninvolved side of the aorta (Figure 5B). The diffuse α-actin expression is most evident in cells that appear outside of the external elastic lamina, suggesting increased migration of SMCs from the outer muscle layer (Figure 5C).

Vascular remodeling that results from increased SMC migration contributes to the development of cardiovascular disease. Changes in the SMC phenotype that allow for increased migration are associated with increased production of extracellular matrix components, particularly hyaluronic acid [17], [18]. We used a specific binding protein for histological detection of hyaluronic acid in the abdominal aorta following AngII infusion. In addition to detecting hyaluronic acid in cells dispersed throughout the AAAs (Figure 5D), areas adjacent to the early AAAs where medial layers remain intact contained cells with concentrated expression of hyaluronic acid (Figure 5D insert). These hyaluronic acid expressing cells appear on the luminal side of the external elastic lamina in the medial smooth muscle layer.

### COX-2 inhibitor treatment alters expression of SMC differentiation markers

**Figure 6:**
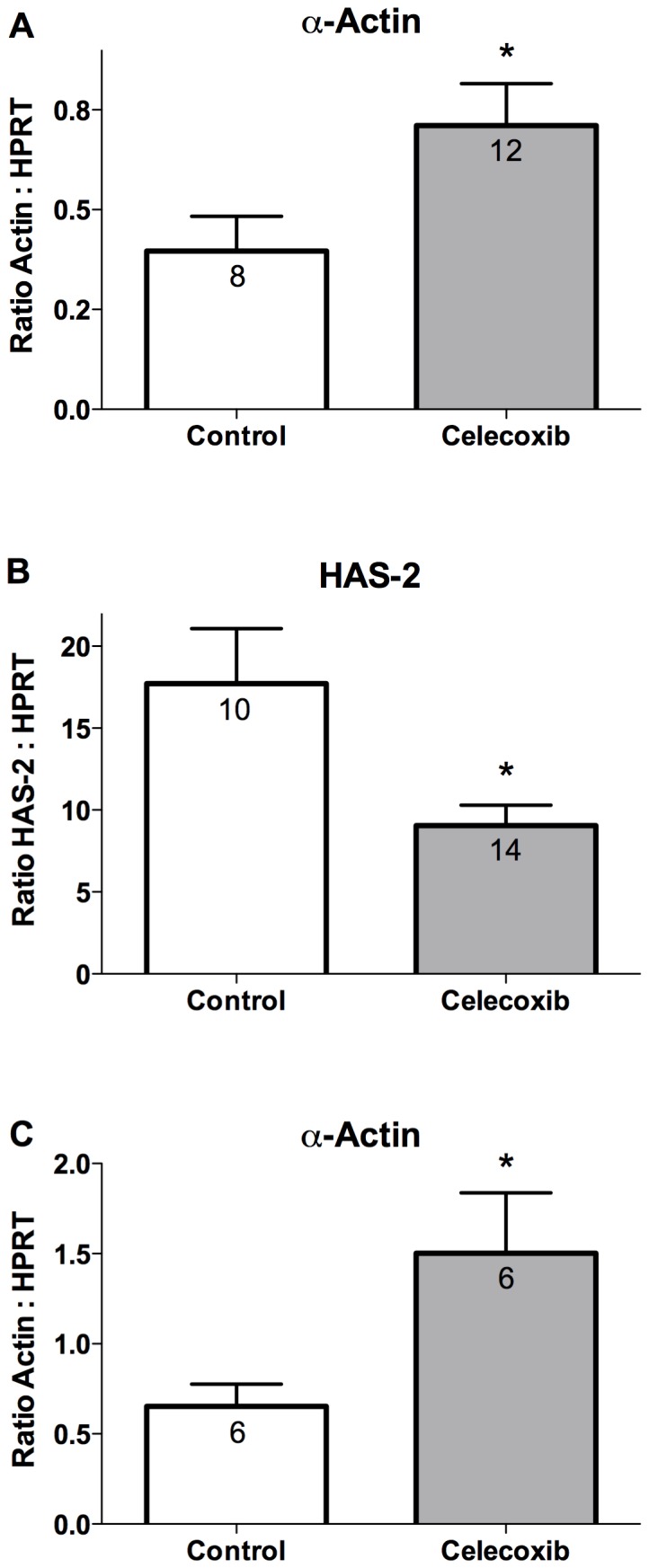
COX-2 inhibitor treatment maintains mRNA expression of markers of differentiated smooth muscle. Real-time PCR quantitation of (A) α-actin or (B) hyaluronic acid synthase-2 mRNA expression in the abdominal aorta. Mice were provided control or celecoxib containing diet beginning 1 week after initiating a 6-week AngII infusion. (C) Quantitation of α-actin mRNA expression in the abdominal aorta of mice provided control or celecoxib containing diet beginning 3 weeks after initiating an 8-week AngII infusion. Expression levels of the gene of interest were normalized to HPRT mRNA levels. Results indicate mean ± SEM and the number of mice in each group is provided within the graphs. *P* values were determined using an unpaired two-tailed *t*-test. * indicates *P*<0.05.

The de-differentiation of SMCs is characterized by reduced mRNA expression of proteins contributing to contractile function and increased gene expression leading to the production of proteins that contribute to a synthetic phenotype [19]. We examined the effect of celecoxib treatment on mRNA expression of the contractile protein α-actin in the abdominal aorta of mice infused with AngII. As compared to mice on control diet, α-actin mRNA expression was significantly increased in mice that received celecoxib treatment that began 1 week following the initiation of a 6-week AngII infusion (Figure 6A). As a marker of SMC de-differentiation, we quantitated the mRNA expression of hyaluronic acid synthase 2 (HAS-2), which is primarily responsible for the synthesis of hyaluronic acid in smooth muscle [20], [21]. As compared to mice on control diet, HAS-2 expression in the abdominal aorta was significantly decreased in mice treated with celecoxib beginning 1 week following the initiation of a 6-week AngII infusion (Figure 6B). We also determined the effect of celecoxib treatment that was started 3 weeks following the initiation of an 8-week AngII infusion. As compared to mice on control diet, α-actin expression in the abdominal aorta was significantly greater in mice receiving celecoxib during the final 5 weeks of the AngII infusion (Figure 6C). These findings indicate that the effectiveness of celecoxib in reducing the progression of AAAs was associated with increased expression of α-actin and reduced HAS-2 expression, characteristics that are consistent with maintenance of a differentiated SMC phenotype.

## Discussion

In the current report, we show that beginning administration of the COX-2 inhibitor celecoxib at an early-stage of AAA development is highly effective at reducing progression of the disease in ApoE-deficient mice. We have previously reported that COX-2 gene expression is increased early in the course of AngII-induced AAA development with significant induction being first detected by 3 days after beginning the AngII infusion [4]. In our current studies which examined the effect of beginning celecoxib treatment 1 week after the start of the AngII infusion, effective COX-2 inhibition would therefore not be expected to occur until after the initial induction of COX-2 expression. This 1 week post AngII infusion celecoxib treatment significantly reduced AAA incidence and severity when analyzed at completion of a 6-week infusion (Figure 1 A–C). Furthermore, the AAA incidence and severity after completion of the celecoxib treatment was similar to that of mice that were not treated with celecoxib and infused with AngII for 1 week (Figure 1 A–C). Therefore, these data suggest that the final 5 weeks of the celecoxib treatment limited progression of the AAA incidence and severity to the level that occurred at the time of beginning the celecoxib treatment.

We also examined the effectiveness of COX-2 inhibition that was started at a late-stage of AAA formation. For this study, celecoxib treatment was started 3 weeks after initiating an 8 week AngII infusion. With the prolonged continuous exposure to AngII for 8 weeks and the potential for an unacceptable level of mortality prior to completion of the study, the AngII infusion rate was reduced from 1 to 0.75 µg/kg/min. In order to determine the level of pathology at the time of beginning the celecoxib treatment, we examined aneurysm formation in mice not receiving celecoxib and infused with AngII for 3 weeks. At this 3 week time-point, the majority of mice showed evidence of an AAA resulting in an overall AAA incidence of 67% (Figure 2A). Beginning celecoxib after 3 weeks of AngII infusion significantly reduced AAA incidence and severity when evaluated after an additional 5 weeks of AngII infusion, as compared to mice on control diet infused with AngII for 8 weeks (Figure 2 A–C). Therefore, although significant AAA pathology was present at the time of beginning celecoxib, COX-2 inhibition effectively reduced further late-stage progression of the disease.

The primary clinical significance for patients developing aortic aneurysms is the increased risk of aortic rupture, which has a 90% mortality rate [22]. Once formed, small AAAs gradually expand over multiple years increasing the risk of eventual rupture and resulting mortality [23]. An effective pharmacological treatment for AAAs must therefore reduce both the progression of aneurysmal remodeling and the resulting aortic rupture. The AngII infusion model is associated with an increase in mortality that results from rupture of the aorta. The incidence of rupture and resulting morality that has been reported in this model using ApoE-deficient mice has varied from 10% to 47% [10], [24]. In the study with the greater levels of mortality, death of the mice occurred throughout the course of the AngII infusion [24]. The wide range of rupture incidence has been suggested to be the result of studies using different aged mice, where mice greater than 6 months of age show a significantly higher mortality than the 10% incidence that occurs in 2-month-old mice [10], [24]. In the current studies which utilized 4 month-old ApoE-deficient mice, the celecoxib-treated group of mice received the COX-2 inhibitor beginning 1 week after the initiation of AngII infusion. The mortality that occurred after the first week of AngII infusion was compared between mice receiving control or celecoxib containing diet. Our findings show that celecoxib treatment beginning 1 week after the initiation of AngII infusion significantly reduced the incidence of mortality during the final 5 weeks of the infusion (Figure 1C). Therefore, COX-2 inhibitor treatment that is started after initiation of the disease is effective for reducing both aneurysmal remodeling and lethal aortic rupture.

We have previously shown that the inhibition of macrophage-dependent inflammation is a prominent feature when COX-2 is inactivated prior to initiating AngII infusion [4]. Therefore, we expected that the effectiveness of COX-2 inhibition for reducing AAAs and aortic rupture when started during the progression-stage of the disease would also correlate with reduced expression of markers of inflammation. We measured mRNA expression of the macrophage marker CD68, the macrophage recruitment chemokine MCP-1, and the pro-inflammatory cytokine TNFα (Figure 3 A–C). However, the effectiveness of COX-2 inhibition for attenuating AAA progression was not associated with reduced expression of these markers of macrophage-dependent inflammation.

AAA development is associated with increased expression of MMPs, particularly MMP-2 and MMP-9 [25]. The increased levels of MMP-9 in human aneurysmal tissue results from expression by infiltrating macrophages whereas increased MMP-2 expression is primarily derived from increased expression by resident cells of the vascular wall [11], [12], [26], [27]. Mouse models of AAAs have shown a similar role for MMP-9 expression being dependent on macrophage infiltration and MMP-2 being derived from mesenchymal cells [1], [28]. Furthermore, the effectiveness of mPGES-1 deficiency in reducing AngII-induced AAAs is not associated with attenuated macrophage-dependent inflammation, but does correlate with reduced activity of MMP-2 and not MMP-9 [5]. Similarly, our current findings show that the effectiveness of COX-2 inhibition for reducing AAA progression was not associated with decreased mRNA expression of MMP-9 or attenuated macrophage-dependent inflammation (Figure 3, A–C, E). In contrast to the effect on MMP-9 expression, celecoxib significantly reduced mRNA expression of MMP-2 (Figure 3D). Increased MMP-2 expression occurs during phenotypic modulation of SMCs and AngII increases the expression of MMP-2 that occurs during SMC de-differentiation [29], [30], [31]. SMC de-differentiation is associated with AAA formation and increased SMC expression of MMP-2 is an early characteristic of the disease [25], [32]. Our finding that celecoxib significantly reduced expression of MMP-2 suggests that COX-2 may contribute to SMC de-differentiation during the progression of AngII-induced AAAs.

An altered level of differentiation of SMCs in the abdominal aorta is an early characteristic of AAA formation. Aortic aneurysm development in humans and animal models of the disease is associated with an altered SMC phenotype resulting in reduced expression of contractile proteins [25]. Mutations in SMC contractile proteins have also been identified as causative factors contributing to inherited forms of aortic aneurysms [33], [34], [35]. Mutations in signaling pathways that cause excessive de-differentiation of aortic SMCs also contribute to inherited forms of aortic aneurysm development [36]. The development of a hematoma in the abdominal aorta is an early event following AngII infusion prior to significant vascular wall remodeling [10]. In the current report, we examined COX-2 expression in the abdominal aorta from mice showing evidence of a hematoma (Figure 4A). As determined by immunohistochemistry, the outer layer of SMCs adjacent to hematomas expressed COX-2 (Figure 4B). COX-2 expression was also concentrated in regions of abdominal aorta outside of a well-defined medial layer adjacent to aneurysmal remodeling (Figure 4 C–E). These regions involved in the remodeling also showed evidence of disorganized cells expressing a variable level of α-actin (Figure 5 A–C). SMCs on the medial side of the external elastic lamina adjacent to AAAs also produced hyaluronic acid (Figure 5D), a characteristic of SMCs following de-differentiation [17]. The effectiveness of celecoxib treatment for attenuating AAA progression was associated with increased mRNA expression of α-actin and decreased expression of hyaluronic acid synthase, characteristics associated with maintenance of a differentiated SMC phenotype (Figure 6). These findings suggest that COX-2 contributes to the de-differentiation of medial SMCs thereby enhancing a migratory phenotype which promotes vascular remodeling during AAA progression.

The expression of COX-2 is critical to the development of AngII-induced AAAs. Our current findings show that COX-2 protein expression in the abdominal aorta is detected in the smooth muscle cell layer of the vessel. Although these findings suggest that the effectiveness of celecoxib in reducing AAA progression may result from inhibiting the activity of COX-2 in SMCs of the vessel, the current studies did not confirm the causative action of COX-2 expressed specifically by the SMCs. Identification of the cell type responsible for the expression of COX-2 contributing to AAA development may be addressed most definitively by future studies utilizing cell-type specific COX-2-deficient mice.

There are some limitations of the current study that should be noted. First, although the effectiveness of celecoxib in reducing AAA expression correlated with increased expression of the SMC differentiation marker α-actin, our findings have not confirmed a causative role for increased SMC differentiation as a mechanism contributing to the effectiveness of COX-2 inhibition. The effect on AAAs that resulted from direct manipulation of SMC differentiation would be required to prove causation of this mechanism. Second, our current findings show that following AngII infusion COX-2 protein expression in the abdominal aorta is detected in the smooth muscle layer of the vessel. Although these findings suggest the importance of COX-2 expressed by SMCs, confirmation of the specific cell type involved would require the use of cell-type specific COX-2-deficient mice. Third, the AngII-induced mouse model has been widely utilized to examine the role of different mechanisms contributing to AAAs. However, there are a variety of animal models of AAA and no single model may accurately reproduce AAA development in humans. Therefore, the significance of our current findings in relation to mechanisms contributing to human AAAs is currently not known.

The chronic use of COX-2 inhibitors has been associated with increased risk of adverse cardiovascular effects in humans. Although the COX-2 inhibitor rofecoxib was withdrawn from the market because of these effects, celecoxib remains available and is widely used in the United States for the treatment of arthritis. While the level of risk from adverse cardiovascular effects that results from celecoxib is controversial, a number of studies suggest that the risk with celecoxib is lower than rofecoxib, and is similar to that of commonly used nonselective COX inhibitors [37], [38], [39]. However, when compared to placebo, the cardiovascular risk of celecoxib has been shown to increase with higher doses, or the presence of preexisting cardiovascular disease [40]. The safety of different doses of celecoxib in patients with preexisting cardiovascular disease is currently being studied in the Prospective Randomized Evaluation of Celecoxib Integrated Safety versus Ibuprofen or Naproxen (PRECISION) trial [41]. Understanding the level of risk of celecoxib in patients with cardiovascular disease is needed before determining the feasibility of future clinical trials for evaluating the therapeutic potential of celecoxib as a treatment for AAAs.
